# Pneumonia, lymphocytes and C-reactive protein are valuable tests for predicting surgical intervention in necrotizing enterocolitis

**DOI:** 10.3389/fped.2023.1231627

**Published:** 2023-07-28

**Authors:** Daiyue Yu, Huirong Yang, Chen Zhong, Kaisi Fan, Guichang Zeng, Mengzhen Zhang, Qianyun Zhao, Jiaming Yang, Liucheng Yang, Kai Wu

**Affiliations:** ^1^ Department of Pediatric Surgery, Zhujiang Hospital, Southern Medical University, Guangzhou, China; ^2^ Department of Pediatric Surgery, Zhongshan Boai Hospital, Zhongshan, China

**Keywords:** neonates, necrotizing enterocolitis (NEC), surgical intervention, risk factor, pneumonia, lymphocytes, C-reactive protein

## Abstract

**Background:**

Necrotizing enterocolitis (NEC) is one of the important causes of neonatal death, and proper timing of operation is of critical significance. This study aimed to explore the high-risk factors for NEC requiring surgical intervention and to provide a reference for its clinical diagnosis and treatment.

**Methods:**

Clinical and radiological evidence of NEC neonates admitted to Zhujiang Hospital of Southern Medical University and Zhongshan Boai Hospital from January 2010 to October 2022 were retrospectively analyzed. Patients were divided into surgical group and conservative group according to whether they underwent surgery or not. Univariate analysis of the clinical data of the two groups was conducted, and multivariate logistic regression analysis was then performed for statistically significant results in the univariate analysis.

**Results:**

267 infants were included in this study, of which 90 patients underwent surgical intervention for NEC and 177 conservation treatment. The univariate analysis showed that the gestational age, pneumonia, leukocytes, lymphocytes, erythrocytes, platelets, C-reactive protein, and blood glucose were statistically significant in the surgical group compared to the conservative group (All *P *< 0.05). Furthermore, the results of multivariate logistic regression analysis showed that compared to the conservative group, patients in the surgical group had a higher proportion of pneumonia (OR = 2.098; 95% CI: 1.030-4.272; *P* = 0.041), lower lymphocyte values (OR = 0.749; 95% CI: 0.588-0.954; *P* = 0.019), and higher C-reactive protein values (OR = 1.009; 95% CI: 1.003-1.016; *P* = 0.004).

**Conclusions:**

Pneumonia, decreased lymphocytes, and elevated C-reactive protein are potential high-risk factors for neonates with NEC requiring surgical intervention and may have potential clinical implications for predicting surgical risk.

## 1. Introduction

Necrotizing enterocolitis (NEC) is an acute necrotizing intestinal disease caused by multiple pathogenic factors in the perinatal period, with abdominal distension, diarrhea, and bloody stools as the main clinical manifestations. NEC progresses rapidly and has a high incidence of peritonitis, shock, and mortality (1–3). The overall incidence of NEC is approximately 0.3–2.4 per 1,000 live births, with 90%–95% occurring in preterm infants whose gestational age was <36 weeks and up to 5%–10% in very low birth weight infants (4). In recent years, the incidence of NEC has been on the rise with the advancement of neonatal diagnosis and treatment technology (4). Due to the lack of efficient early diagnostic tools and rapid disease progression, NEC remains one of the most common causes of death in newborns. Preterm infants weighing less than 1,500 g have a mortality rate of 20%–30% (3).

Surgical intervention is an important method of treatment for neonates with NEC. Abdominal radiography suggestive of gastrointestinal perforation and deterioration after conservative treatment are indications for surgery in NEC (5). However, the optimal timing of surgical intervention for neonates with NEC remains challenging (6–8). More than half of the infants with NEC requiring surgical intervention lack definite surgical signs (9). Therefore, the identification of simple and effective early predictors of surgical intervention in neonates with NEC is of great significance to improve the long-term survival of NEC patients.

In this study, the clinical data, such as general blood examinations, ultrasensitive C-reactive protein (CRP), and blood glucose of NEC patients admitted to Zhujiang Hospital of Southern Medical University and Zhongshan Boai Hospital were analyzed. We aimed to explore the high-risk factors for neonates with NEC requiring surgical intervention and to identify these potential factors to provide a clinical reference for early prevention and reduction of surgical risks.

## 2. Materials and methods

### 2.1. Study population

A case-control study of all neonates diagnosed with NEC admitted to the neonatal intensive care unit of Zhujiang Hospital of Southern Medical University and Zhongshan Boai Hospital from January 2010 to October 2022.

The diagnosis of NEC is primarily based on clinical presentation (i.e., feeding intolerance, abdominal distension, occult or gross blood, abdominal tenderness, abdominal wall erythema/discoloration or abdominal mass) and radiological evidence suggestive of NEC. Within the included patients who met the inclusion criteria, newborns were divided into conservation group (patients with NEC conservative treatment): 177 patients, and surgery group (patients with NEC conservative treatment failed): 90 patients. When the neonatologist suspects a neonate with NEC, CRP, blood routine examination and biochemical examination were carried out. Blood samples were collected after the children were diagnosed with NEC and before surgery.

Surgical intervention was defined as performance of exploratory laparotomy or laparoscopy. The indication of surgical intervention is intestinal perforation (leading to pneumoperitoneum) or clinical deterioration after conservative treatment (7, 10). In the surgical group, perforation was diagnosed according to macroscopic and pathological findings. Patients in the surgery group were excluded if they were diagnosed with spontaneous intestinal perforation during exploratory laparotomy and pathological examination. Diagnosis of pneumonia and congenital heart disease combined with symptoms, signs, x-ray and ultrasound. All inspection and examination results are obtained within 48 h of diagnosis of NEC. Moreover, all infants were diagnosed by two neonatologists, and senior pediatric surgeons determined the surgical indications and performed the operations.

### 2.2. Data collection

Details of each patient were obtained from hospital's electronic system. Information was entered using a Case Report Form (CRF) designed in Microsoft Word. And then the data was organized using Microsoft Excel. The clinical data of each subject during hospitalization was collected, including (1) patient demographics such as gender, age, gestational age (GA), birth weight, blood type, etc; (2) perinatal factors like maternal history, maternal age, delivery method, intrauterine infection, feeding method, history of ischemia and hypoxia, etc; (3) laboratory examination including erythrocytes, hemoglobin, white blood cells, platelets, C-reactive protein (CRP), blood glucose, etc; (4) complications: congenital heart disease, pneumonia, shock, etc. This study was approved by the Medical Ethics Committee, Zhujiang Hospital of Southern Medical University (NO. 2023-KY-035-01) and Zhongshan Boai Hospital (NO. KY-2023-02-05). Besides, the study strictly complied with the principles of the Declaration of Helsinki.

### 2.3. Statistical analysis

All data were statistically analyzed using SPSS 25.0 software. Normal distribution data were expressed as mean ± standard deviation (Mean ± SD), and comparisons between groups were made using the independent samples *t*-test. Non-normal distribution data were expressed as quartile [Q2(Q1,Q3)], and comparisons between groups were made using the Mann-Whitney *U*-test. Count data sets were expressed as cases (%), and comparisons between groups were made using the *χ*^2^-test or Fisher's exact probability method. Multi-factor logistic regression analysis was performed for factors that were statistically significant in the univariate analysis. A *P* value <0.05 was considered statistically significant.

## 3. Results

### 3.1. General characteristics of NEC patients

A total of 267 infants were included in this study, of which 90 underwent surgical intervention for NEC (Surgery group) and 177 received conservative treatment (Conservation group). Among them, 169 (63.3%) were male and 98 (36.7%) were female.175 (65.5%) were preterm, and 92 (34.5%) were full-term.170 (63.7%) were low birth weight infants(<2,500 g), and 97 (36.3%) were normal birth weight infants (≥2,500 g). As it is shown in Table 1, the gestational age in the surgical group was lower than that in the conservative group (*P *= 0.009), and the incidence of pneumonia was higher than that in the conservation group (*P *= 0.002). There was no statistically significant difference between the two groups in terms of age at diagnosis of NEC, gender, birth weight, and whether congenital heart disease was combined (*P *> 0.05).

**Table 1:** The clinical characteristics of NEC patients.

Variables	Conservation group (*n* = 177)	Surgery group (*n* = 90)	*P* value
Age at NEC diagnosis (days), Q2 [Q1, Q3]	5 (2.00, 9.25)	6 (2.00, 11.00)	0.248
Gestational age(weeks), Q2 [Q1, Q3]	35.21 (32.29, 38.04)	33.57 (30.00, 38.00)	0.009
Gender (*n*/%)			0.279
Male	108 (61.02)	61 (67.78)	
Female	69 (38.98)	29 (32.22)	
Premature birth (*n*/%)			0.172
<37 weeks	111 (62.71)	64 (71.11)	
≥37 weeks	66 (37.29)	26 (28.89)	
Low birth weight (*n*/%)			0.125
<2,500 g	107 (60.45)	63 (70.00)	
≥2,500 g	70 (39.55)	27 (30.00)	
Congenital heart disease (*n*/%)			0.861
Yes	132 (74.58)	68 (75.56)	
No	45 (25.42)	22 (24.44)	
Pneumonia (*n*/%)			0.002
Yes	83 (46.89)	60 (66.67)	
No	94 (53.11)	30 (33.33)	

### 3.2. Perinatal characteristics of NEC patients

There were no statistically significant differences in maternal age, pregnancy status, delivery method, feeding method, history of intrauterine infection, and history of ischemia and hypoxia between the two groups (all *P* > 0.05) (Table 2).

**Table 2:** The perinatal features of NEC patients.

Variables	Conservation group (*n* = 177)	Surgery group (*n* = 90)	*P* value
Maternal age (years)	29.89 ± 5.69	30.59 ± 6.11	0.404
Pregnancy (*n*/%)			0.289
Single	145 (81.92)	76 (84.44)	
Twins	32 (18.08)	13 (14.44)	
Triplets and above	0 (0.00)	1 (1.11)	
Delivery method (*n*/%)			0.898
Vaginal delivery	88 (49.72)	44 (48.89)	
Cesarean section	89 (50.28)	46 (51.11)	
Feeding method			0.155
Breast milk	93 (52.54)	39 (43.33)	
No breast milk	84 (47.46)	51 (56.67)	
History of intrauterine infection (*n*/%)			0.658
Yes	13 (7.34)	8 (8.89)	
No	164 (92.66)	82 (91.11)	
History of ischemia and hypoxia (*n*/%)			0.282
Yes	59 (33.33)	36 (40.00)	
No	118 (66.67)	54 (60.00)	

### 3.3. Predictive value of blood test

Compared with the conservative group, neonates in the surgical group had lower leukocytes (*P *= 0.001), lymphocytes (*P* < 0.001), erythrocytes (*P* = 0.004), and platelets (*P* = 0.039), and higher CRP value (*P* < 0.001) and blood glucose (*P* = 0.044). The differences were all statistically significant. In contrast, the differences in hemoglobin values and neutrophil values between the two groups were not statistically significant (*P* > 0.05) (Table 3).

**Table 3:** The predictive value of blood test.

Blood test parameters	Conservation group (*n* = 177)	Surgery group (*n* = 90)	*P* value
Leukocyte [10^9^/L, Q2 (Q1, Q3)]	9.93 (6.19, 14.04)	6.32 (3.16, 12.42)	0.001
Neutrophil [10^9^/L, Q2 (Q1, Q3)]	4.53 (2.80, 7.86)	3.77 (1.50, 8.93)	0.091
Lymphocyte [10^9^/L, Q2 (Q1, Q3)]	2.62 (1.59, 4.13)	1.62 (0.86, 2.59)	<0.001
Erythrocyte [10^12^/L, Mean (SD)]	3.77 ± 0.92	3.48 ± 0.70	0.004
Hemoglobin [g/L, Mean (SD)]	128.18 ± 29.39	120.62 ± 28.00	0.053
Platelet [10^9^/L, Q2 (Q1, Q3)]	217.50 (131.25, 300.50)	168.00 (109.50, 261.75)	0.039
C-reactive protein [mg/L, Q2 (Q1, Q3)]	5.20 (0.86, 27.55)	37.09 (4.77, 99.47)	<0.001
Glucose [mmol/L, Q2 (Q1, Q3)]	4.10 (3.00, 5.23)	4.89 (3.09, 7.28)	0.044

### 3.4. Multivariate logistic regression analysis

Based on the results of the univariate analysis, statistically significant variables were subjected to multivariate logistic regression analysis. Gestational age, low birth weight, pneumonia, history of ischemia and hypoxia, history of intrauterine infection, leukocytes, lymphocytes, erythrocytes, platelets, CRP, and blood glucose were used as independent variables, and whether the neonates with NEC underwent surgical intervention was used as the dependent variable for the multivariate logistic regression analysis. The results of multifactorial statistics showed that in comparison to the conservative group, the incidence of pneumonia was higher (OR = 2.098; 95% CI: 1.030-4.272; *P* = 0.041), the CRP values were higher (OR = 1.009; 95% CI: 1.003-1.016; *P* = 0.004), and lymphocyte values were lower (OR = 0.749; 95% CI: 0.588-0.954; *P* = 0.019) in the surgical group, suggesting that pneumonia and CRP are independent risk factors for surgical intervention in neonates with NEC and lymphocytes is a potentially protective factor, with statistically significant differences (Table 4).

**Table 4:** Multivariate logistic regression analysis results of NEC patients.

Risk factors	*β*	Wald *χ*^2^ value	*P* value	OR (95% confidence interval)
Pneumonia	0.741	4.167	0.041	2.098 (1.030–4.272)
Lymphocyte	−0.289	5.475	0.019	0.749 (0.588–0.954)
C-reactive protein	0.009	8.491	0.004	1.009 (1.003–1.016)
Gestational age	−0.107	1.839	0.175	0.899 (0.771–1.049)
Leukocyte	0.020	0.594	0.441	1.020 (0.970–1.072)
Erythrocyte	−0.398	3.534	0.060	0.672 (0.444–1.017)
Platelet	0.002	3.336	0.068	1.002 (1.000–1.005)
Glucose	0.037	0.474	0.491	1.037 (0.935–1.151)

## 4. Discussion

The incidence of NEC is extremely high in preterm infants, with rapid progression and high mortality (3). With advances in neonatal intensive care technology, the survival rate of premature and low birth weight infants has increased in the past few years. This has been followed by a gradual increase in the incidence of NEC. Internal treatment is effective for most cases of NEC, which includes intestinal decompression, bowel rest, intravenous fluid administration, and antibiotic therapy. A crucial part of internal treatment is close observation using serial abdominal physical examinations to monitor any deterioration in condition and abdominal radiographs to monitor for perforation, if the condition changes surgery is needed. Nearly 30%–50% of neonates with NEC require surgical intervention (11). However, there are no criteria for accurately extrapolating the timing of the operation (12). Therefore, the identification of high-risk factors for surgical intervention and timely surgical treatment are essential to improve the prognosis of neonates with NEC.

In our study, we found that gestational age, pneumonia, leukocytes, lymphocytes, red blood cells, platelets, CRP and blood sugar levels were correlated with the risk of surgical intervention in NEC. Through multivariate correction analysis, we found that CRP, lymphocytes, and pneumonia were independent risk factors for the need for surgical intervention in NEC neonates.

When patients are exposed to infection or tissue damage, CRP is synthesized by the liver, and then interacts with the immune system to participate in the defense against infection. Studies have reported that CRP can reflect the severity of NEC neonates (13, 14). Miner et al. (15) demonstrated that NEC can provoke a systemic inflammatory response syndrome (SIRS), and a higher CRP level might predict a poor prognosis. Pourcyrous et al. (16) suggested that an elevated CRP level can predict whether NEC premature neonates require surgical intervention. It has also been reported that the combination of leukocyte, platelet and C-reactive protein can predict whether NEC patients need surgical intervention (17). In this study, we also found that CRP in the surgical group was significantly higher than that in the non-surgery group, and the difference was statistically significant after correction by multivariate logistic regression analysis, which was consistent with the conclusions of previous studies. Our results suggest that elevated CRP may be a potential independent risk factor for surgical intervention in neonates with NEC.

Lymphocyte is a kind of leukocyte, produced by the human lymphatic organ, with immune recognition and response function, its elevation represents the body infection or inflammatory response. Previous studies have found that platelet-to-lymphocyte ratio (PLR) and neutrophil-to-lymphocyte ratio (NLR) can be used to predict the severity of NEC (18, 19), but lymphocytes have not been analyzed separately. In this study, lymphocytes in the surgical group were significantly lower than that in the conservative group, suggesting that lymphocytes were a protective factor in NEC. Lymphocytes are an important component of the immune response. When NEC deteriorated, the body subsequently undergoes a severe inflammatory storm that leads to lymphocyte depletion (12). Another reason is that in severe infections, bacteria and toxins can stimulate the specific immune activation of lymphocytes, and the body's immune imbalance leads to an increase in lymphocyte apoptosis, which in reverse reduces the number of lymphocytes (20, 21). Our results also confirmed that lymphocytes in NEC neonates requiring surgical intervention were lower than those of neonates receiving medical management.

NEC is a multifactorial disease, and studies have proved that history of asphyxia and hypoxia are high-risk factors for NEC (22). Lack of oxygen can exacerbate intestinal disorders and aggravate disease progression (23). Willis et al. (24) proposed that there is an “entero-pulmonary axis” between the gut and the lungs of NEC patients. Firstly, they found an increased incidence of NEC in neonates with hypoxemia and pulmonary infection; by contrast, NEC can cause multi-organ dysfunction and abdominal distention, reducing the rate of gas exchange in the lungs. Increasing self-ventilation when demand exceeds supply can in turn intensify lung damage. Jia et al. (25) suggested that premature infants with NEC were more likely to develop lung injury than full-term infants. In our study, pneumonia in the surgical group was more common than in the conservative group, indicating that neonates with serious lung diseases are more prone to develop severe NEC and require surgical intervention.

In conclusion, CRP and pneumonia are potential independent risk factors for surgical intervention in neonates with NEC, while lymphocytes are protective factors. Attention to the presence of pneumonia, changes in CRP, and lymphocyte levels in neonates with NEC, combined with indicators such as clinical manifestations, is potentially significant in predicting the need for surgical intervention and their prognosis. Nevertheless, there are still some limitations in this study. Firstly, as a retrospective study, the level of evidence in this study was not sufficiently high, we still need to conduct multi-center prospective randomized controlled studies in the future. In addition, the risk factors identified in this study cannot be used independently to assess the severity of NEC and the risk of surgery. It is still necessary to make a comprehensive judgment by the department of neonatology and neonatal surgeons in conjunction with the clinical manifestations and other examination results of the children.

## Data Availability

The original contributions presented in the study are included in the article/Supplementary Material, further inquiries can be directed to the corresponding authors.
